# Analysis of the Increase of Vascular Cell Adhesion Molecule-1 (VCAM-1) Expression and the Effect of Exposure in a Hyperbaric Chamber on VCAM-1 in Human Blood Serum: A Cross-Sectional Study

**DOI:** 10.3390/medicina58010095

**Published:** 2022-01-08

**Authors:** Katarzyna Van Damme-Ostapowicz, Mateusz Cybulski, Mariusz Kozakiewicz, Elżbieta Krajewska-Kułak, Piotr Siermontowski, Marek Sobolewski, Dorota Kaczerska

**Affiliations:** 1 Department of Health and Caring Sciences, Faculty of Health and Social Sciences, Western Norway University of Applied Sciences, Svanehaugvegen 1 Str., 6812 Førde, Norway; 2 Department of Integrated Medical Care, Faculty of Health Sciences, Medical University of Białystok, Skłodowskiej-Curie 7A Str., 15-096 Białystok, Poland; mateusz.cybulski@umb.edu.pl (M.C.); elzbieta.krajewska-kulak@umb.edu.pl (E.K.-K.); 3 Division of Biochemistry and Biogerontology, Department of Geriatrics, Nicolaus Copernicus University in Torun, L. Rydygier Collegium Medicum in Bydgoszcz, Dębowa 3 Str., 85-626 Bydgoszcz, Poland; markoz@cm.umk.pl; 4 Department of Submarine Work Technology, Faculty of Mechanical and Electrical Engineering, Polish Naval Academy, Śmidowicza 69 Str., 81-127 Gdynia, Poland; p.siermontowski@amw.gdynia.pl; 5 Department of Quantitative Methods, Faculty of Management, Rzeszów University of Technology, Powstańców Warszawy 8 Str., 35-959 Rzeszów, Poland; msobolew@prz.edu.pl; 6 Department of Physiotherapy and Health Sciences, Faculty of Dietetics, Gdańsk College of Health, Pelplińska 7 Str., 80-335 Gdańsk, Poland; dorotakaczerska@tlen.pl

**Keywords:** atmosphere exposure chambers, diving physiology, decompression

## Abstract

*Background and Objectives:* Vascular cell adhesion molecule-1 (VCAM-1) was identified as a cell adhesion molecule that helps to regulate inflammation-associated vascular adhesion and the transendothelial migration of leukocytes, such as macrophages and T cells. VCAM-1 is expressed by the vascular system and can be induced by reactive oxygen species, interleukin 1 beta (IL-1β) or tumor necrosis factor alpha (TNFα), which are produced by many cell types. The newest data suggest that VCAM-1 is associated with the progression of numerous immunological disorders, such as rheumatoid arthritis, asthma, transplant rejection and cancer. The aim of this study was to analyze the increase in VCAM-1 expression and the impact of exposure in a hyperbaric chamber to VCAM-1 levels in human blood serum. *Materials and Methods*: The study included 92 volunteers. Blood for the tests was taken in the morning, from the basilic vein of fasting individuals, in accordance with the applicable procedure for blood collection for morphological tests. In both groups of volunteers, blood was collected before and after exposure, in heparinized tubes to obtain plasma and hemolysate, and in clot tubes to obtain serum. The level of VCAM-1 was determined using the immunoenzymatic ELISA method. *Results*: The study showed that the difference between the distribution of VCAM-1 before and after exposure corresponding to diving at a depth of 30 m was at the limit of statistical significance in the divers group and that, in most people, VCAM-1 was higher after exposure. Diving to a greater depth had a much more pronounced impact on changes in VCAM-1 values, as the changes observed in the VCAM-1 level as a result of diving to a depth of 60 m were statistically highly significant (*p* = 0.0002). The study showed an increase in VCAM-1 in relation to the baseline value, which reached as much as 80%, i.e., VCAM-1 after diving was almost twice as high in some people. There were statistically significant differences between the results obtained after exposure to diving conditions at a depth of 60 m and the values measured for the non-divers group. The leukocyte level increased statistically after exposure to 60 m. In contrast, hemoglobin levels decreased in most divers after exposure to diving at a depth of 30 m (*p* = 0.0098). *Conclusions*: Exposure in the hyperbaric chamber had an effect on serum VCAM-1 in the divers group and non-divers group. There is a correlation between the tested morphological parameters and the VCAM-1 level before and after exposure in the divers group and the non-divers group. Exposure may result in activation of the endothelium.

## 1. Introduction

Vascular cell adhesion molecule-1 (VCAM-1) is an endothelial cell adhesion factor. It is expressed on endothelium activated by cytokines, but may also occur in a soluble form in serum [1]. VCAM-1 was identified as a cell adhesion molecule that helps regulate inflammation-associated vascular adhesion and the transendothelial migration of leukocytes, such as macrophages and T cells. VCAM-1 is expressed by the vascular system and can be induced by reactive oxygen species, interleukin 1 beta (IL-1β) or tumor necrosis factor alpha (TNFα), which are produced by many cell types. The newest data suggests that VCAM-1 is associated with the progression of numerous immunological disorders, such as rheumatoid arthritis, asthma, transplant rejection and cancer [1]. Homeostasis is a necessary condition for health and the proper functioning of the body, and hence, diseases result from disturbances in mechanisms that maintain homeostasis [2]. Commercial saturation divers work in high-pressure environments, in which their bodies must acclimatize to a variety of physiological stress factors [3].

Research shows that intercellular adhesion molecule-1 (ICAM-1) and VCAM-1 adhesive molecules are potential markers of changes in the endothelium [4]. These molecules are of great interest both in order to understand the mechanisms of their action and their usefulness in the diagnosis and treatment of diseases [4,5]. Brubakk et al. [6], in their studies on vesicle formation and endothelial function in human and animal models, showed a decrease in the arterial endothelial function after a single dive using air.

The researchers [7,8,9] indicated that the endothelium was sensitive to oxidative stress and the shear rate, leading to vascular remodeling and a release of micro molecules. According to Freyssinet [9], endothelial microparticles are constantly shed into the circulation of healthy individuals and have been shown to be elevated in many diseases, most notably those characterized by endothelial dysfunction. This was supported by Horstman et al. [10].

It would be interesting to deepen the knowledge of the role of the VCAM-1 biomarker in the human body during decompression. The aim of this study was to analyze the increase in VCAM-1 expression and the impact of exposure in a hyperbaric chamber to the VCAM-1 level in human blood serum.

The research problem was to answer the following questions:

1. Does exposure in the hyperbaric chamber affect the VCAM-1 level in the divers group and non-divers group (a group who stayed in the same chamber for the same time period and breathed in the same pattern with the same breathing mix to best reflect the effect of the same pressure during exposure and decompression)?
2. Is there a correlation between the blood cell counts (BCC) and VCAM-1, before and after exposure in the chamber, in the divers group?

The following hypotheses were formulated:

1. Exposure in a hyperbaric chamber has an effect on the serum VCAM-1 level in the divers group and the non-divers group.
2. There is a correlation between tested BCC and VCAM-1 before and after exposure in the chamber in the divers group.

## 2. Materials and Methods

### 2.1. Design of the Study

The cross-sectional study involved four exposures. Short-term simulated hyperbaric air exposures corresponding to diving at a depth of 30 and 60 m were carried out. The exposure corresponding to a 60 m dive was chosen because it was the maximum allowable depth for a dive using air as a breathing mixture, and 30 m as half the maximum depth. Air was used for breathing in the hyperbaric chamber during dives. This was an experimental chamber complex DGKN 120 belonging to the Department of Underwater Works Technology of the Naval Academy in Gdynia. It consists of 3 chambers located at the same level: dry, wet and transient. In a dry chamber, where the study was carried out on short-term exposures, there may be 7 people, with longer ones—4, and with saturated ones—2. The maximum working pressure is 120 m of water column, i.e., 12 at or 13 ata. Additional inhalers (beeps) allow you to breathe, e.g., oxygen in a different atmosphere in the chamber. Pressure unit converters were the following: 760 mmHg = 760 tracks ~ 1 atm. = 1.033227 at. = 1.01325 N/m^2^ = 1.01325 Pa = 14.69 psi.

**Figure 1:**
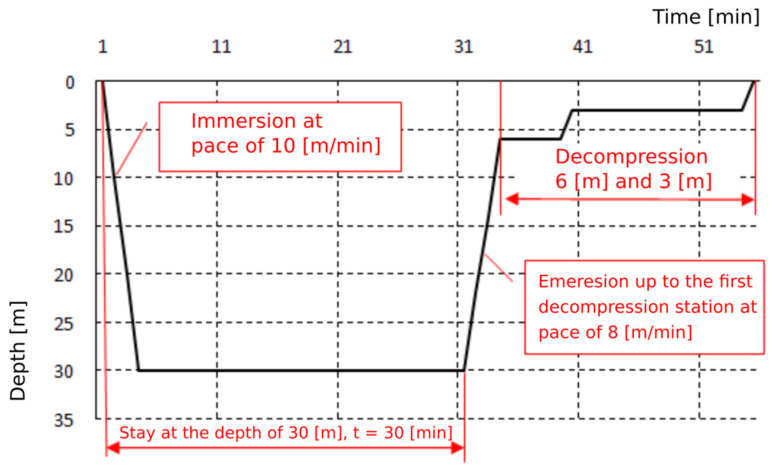
Pressure exposure profile 30 m/30 min.

**Figure 2:**
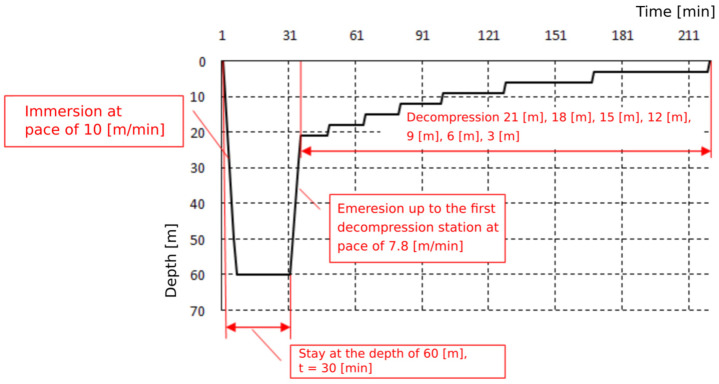
Pressure exposure profile 60 m/30 min.

Exposures were based on the Naval Table for the decompression and recompression of divers (Table 1). Exposures were carried out by compressing the subjects in a hyperbaric chamber to a pressure of 400 kPa, corresponding to a dive at a depth of 30 m, and to a pressure of 700 kPa, corresponding to a dive at a depth of 60 m. This pressure was maintained for 30 min. The entire time of exposure to 4 atm was 1 h, and to 7 atm—2 h. The plateau of both exposures was 30 min. The pressure exposure profiles are shown on Figure 1 and Figure 2. Exposures were performed at the Department of Underwater Works Technology of the Naval Academy in cooperation with the Department of Maritime and Hyperbaric Medicine of the Military Medical Institute in Gdynia. Exposures were carried out by a qualified physician and a technical employee of the Department of Underwater Works Technology of the Naval Academy in Gdynia.

### 2.2. Characteristics of Subject Population

Volunteers also completed a questionnaire, providing information concerning their age, sex, place of residence, education, seniority, type of work, type of physical exertion, smoking status, coffee consumption and a self-assessment of their physical condition. A total of 45 professional divers volunteered to participate in the study. They were subjected to hyperbaric exposure in a pressure chamber. A total of 47 volunteers—non-divers who had never been subjected to hyperbaric exposure—were also included. The non-divers group stayed in the same chamber for the same time period and breathed in the same pattern with the same (identical) breathing mixture to best reflect the effect of the same pressure during exposure and decompression. The non-divers group, who were not exposed, sat in the in the same chamber, with the same temperature and lighting conditions and breathed the same breathing mixture.

Criteria for inclusion in the divers group were professionally active diver, mentally and physically healthy and aged from 24 to 55 years. Exclusion criteria were respiratory tract infection, age under 24 and over 55 years, using intoxicating drugs, using any other medications and resignation from participation in the study. Ultimately, 18 people participated in the study. Inclusion criteria for the non-divers group were non-divers, who had never been subjected to hyperbaric exposure before, mentally and physically healthy and aged 24 to 55 years. Exclusion criteria were respiratory tract infection, aged under 24 and over 55, using intoxicating drugs, using any other medications and resignation from participation in the study Ultimately, 14 people participated in the study.

All the people who participated in the study breathed air and were not subjected to physical exertion.

Basic information about the divers group (N = 18) and the non-divers group (N = 14) is presented in Table 2. There were statistically significant differences between the sex (*p* = 0.0391), type of work (*p* = 0.0436) and physical effort (*p* = 0.0043) between the groups. In the divers group, manual workers predominated. The descriptive statistics presented below show that both groups were completely comparable in terms of age and occupational seniority.

### 2.3. Methods

Blood for the tests was taken in the morning, from the basilic vein of fasting individuals, in accordance with the applicable procedure for blood collection for morphological tests. Tests were performed by a certified medical analytical laboratory. VCAM-1 measurements were performed with serum and BCC with plasma.

In both groups, blood was collected at the same time, before and after exposure, to heparin anticoagulant tubes to obtain plasma and hemolysate and to clot tubes to obtain serum. The level of VCAM-1 was determined using the immunoenzymatic ELISA method, with the DIACLONE kit.

### 2.4. Procedural and Ethical Considerations

The study was performed from September 2018 to June 2019 and the study obtained ethical approval from the Bioethics Committee of the Medical University in Bialystok, Poland (R-I-002/237/2015). Members of the research team provided oral and written information about the study. Subjects gave their informed consent for participation in the study. Each participant received written and oral information about the possibility of withdrawing from the study at any time and without any consequences. The research conformed with the Good Clinical Practice guidelines, and the procedures were in accordance with the principles of the 1975 Declaration of Helsinki, as revised in 2000, and with the ethical standards of the institutional committee on human experimentation.

### 2.5. Statistical Analysis

To present listings as elements of the description of both groups, summary tables contain numbers and percentages, and for age and occupational seniority—means (M) ± standard deviations (SD); the *p*-value was calculated using the chi-square test of independence (for comparison of percentage structure) or the Mann–Whitney U test (for comparison of numerical values—age and occupational seniority of the subjects).

Selected numerical characteristics of the examined parameters were determined: arithmetic mean (M), median (Me), the highest (maximum) and the lowest (minimum) value and standard deviation (SD).

A test of the statistical significance of the relationship under study was performed. For all statistical analyses, the significance level was set at *p* < 0.05.

Additionally, information on the 95% confidence interval for the average VCAM-1 level measured in the four tested situations is presented. The normality of VCAM-1 level distribution in various tested situations was assessed using the Shapiro–Wilk test. The statistical significance of differences in the distribution of VCAM-1 before and after exposure was analyzed. As the measurements were made on the same group of divers, the Wilcoxon test was used for the analysis. Results were graphically illustrated using scatter plots. The Mann–Whitney U test was used to compare the distribution of VCAM-1 among non-divers (in various tested situations). The difference between the results obtained in both groups was assessed using the Mann–Whitney U test. The Wilcoxon test was also used to examine the significance of changes between tests performed before and after exposure.

Spearman’s rank correlation coefficient (*r*_S_) was used to assess the strength of relationships between the BCC and VCAM-1.

## 3. Results

There were no significant differences between the divers and non-divers groups in terms of coffee consumption, smoking status or self-assessment of physical condition.

The *p*-value calculated for “coffee consumption” was *p* = 0.3365, for “smoking status”—*p* = 0.7876 and for “self-assessment of physical condition”—*p* = 0.0842 (Table 3).

The table below (Table 4) provides information on the 95% confidence intervals for the average level of VCAM-1 in the four tested situations.

The difference between the VCAM-1 distribution before and after the exposure with a diving depth of 30 m was on the limit of statistical significance (*p* = 0.0582). In most people, VCAM-1 after exposure was higher, on average, by about 1.5 ng/mL (but a decrease in the VCAM-1 level was also noted in some people). Diving to a greater depth had a much more pronounced impact on the changes in the VCAM-1 value. In all the subjects, VCAM-1 increased after exposure by at least 0.8, and at most by 24.2 ng/mL. On average, the change was about 5.9 ng/mL, although the average was somewhat overestimated by the quite outlying peak value of VCAM-1 growth of 24.2. Therefore, a median of 4.2 ng/mL may be a better measure of the average level of VCAM-1 changes after a dive (Table 5).

The changes in VCAM-1 as a result of diving to a depth of 60 m were highly statistically significant (the *p*-value determined using the Wilcoxon test was 0.0002).

**Figure 3:**
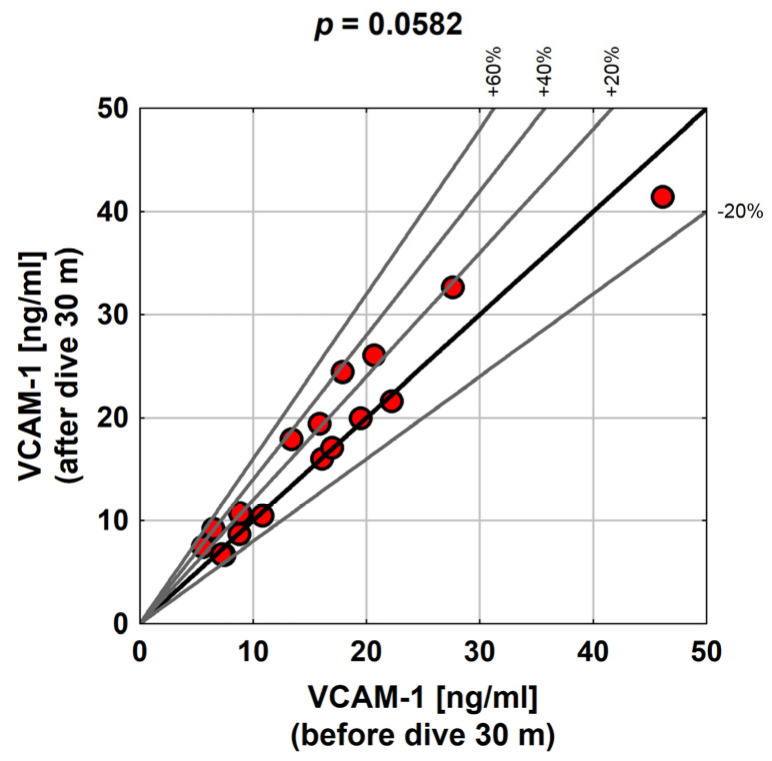
Distribution of VCAM-1 before and after exposure to a 30 m dive in the study group.

As seen in the chart below, the relative increase in VCAM-1 in some people was as high as 60% of the initial value (Figure 3).

**Figure 4:**
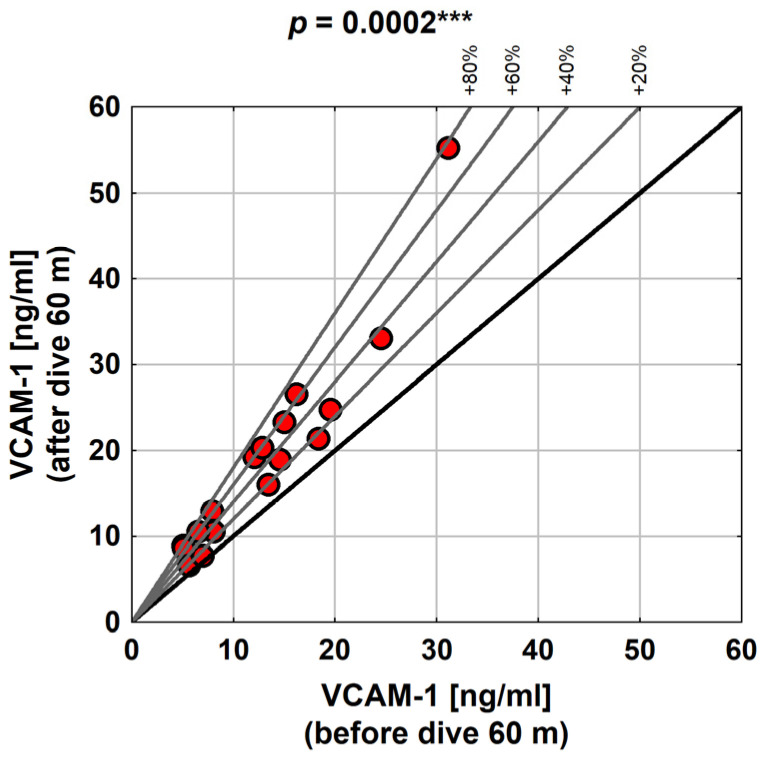
VCAM-1 values with diving to 60 m in the study group. Abbreviations: ***—*p* < 0.001.

As presented in the graph below (Figure 4), the relative increase in VCAM-1 over the baseline was as high as 80% (i.e., in some subjects, VCAM-1 was almost twice as high after diving).

The distribution of results obtained before and after exposure to the conditions corresponding to diving at a depth of 30 m did not differ in a statistically significant way from the distribution of results in the non-divers group.

Statistically significant differences existed between the results obtained after exposure to diving conditions at a depth of 60 m and the values measured for the non-divers group (*p* = 0.0494; Table 6).

**Figure 5:**
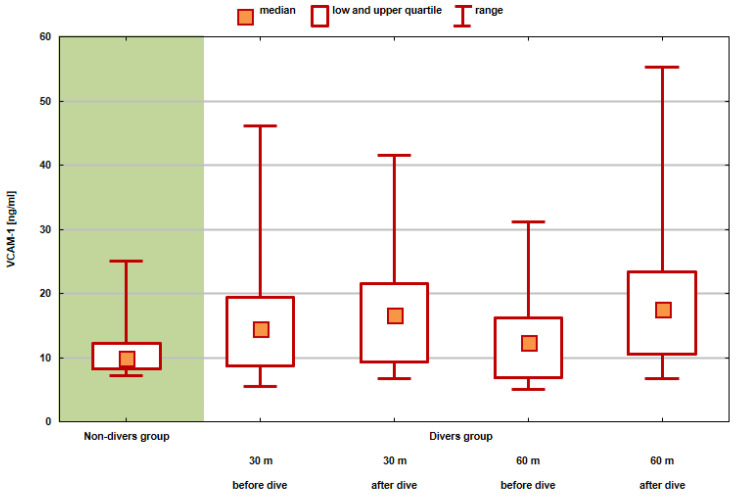
Values of position statistics of VCAM-1 distribution in the compared groups and test series.

The graph (Figure 5) shows the values of position statistics of the VCAM-1 distribution in the compared groups and test series.

The leukocyte count increased in a statistically significant manner after exposure to a 60 m dive. However, exposure to the conditions corresponding to diving at a depth of 30 m did not affect the unequivocally directed change in the leukocyte count. Longer exposure results in greater tissue saturation with gases (among others with nitrogen) during decompression, which lasts significantly longer; in this case, many more microbubbles are formed, which activate the immune system. The hemoglobin level decreased in most divers after exposure to a 30 m dive (*p* = 0.0098). Detailed data presenting the distribution of leukocytes and hemoglobin counts in individual tests, showing the significance of changes between the tests completed before and after exposure, are presented in Table 7.

The results of BCC after the exposure of the subjects in the divers group to conditions corresponding to a 30 m dive were not correlated with the VCAM-1 values. All the correlation coefficients were statistically insignificant (*p* > 0.05). Those few correlations that were close to the level of statistical significance, and also in a single case that was statistically significant, occurred between BCC and VCAM-1 after exposure to conditions corresponding to a 60 m dive.

These relationships were as follows:

- Relationships between the neutrophil count and VCAM-1—the higher the level of neutrophils was, the lower the value of VCAM-1 was (*p* = 0.0456, R = −0.51);
- A similar relationship existed between the leukocyte count and VCAM-1 (it was slightly weaker and only close to the level of statistical significance);
- VCAM-1 was higher in subjects with a higher lymphocyte ratio (R = 0.44)—this correlation was close to the level of statistical significance (*p* = 0.0848).

Changes in the BCC and VCAM-1 level after exposure to conditions corresponding to a 30 m dive were not statistically significantly correlated with each other.

More statistically significant relationships existed between the changes in BCC and the changes in VCAM-1 after exposure to the conditions corresponding to a 60 m dive.

There were the following relationships:

- Higher increases in VCAM-1 were associated with a greater increase (in some cases, a smaller decrease) in the mean corpuscular hemoglobin (MCH) and the mean corpuscular hemoglobin concentration (MCHC)—these were relationships of average strength and statistical significance;
- Quite a strong correlation occurred between the changes in the neutrophil counts and the changes in VCAM-1—the greater the increase in the number of neutrophils was, the smaller the increase in VCAM-1 was;
- In turn, the increase in the lymphocyte count after exposure to the conditions corresponding to a 60 m dive was correlated with the increase in VCAM-1 (R = 0.54; *p* = 0.0304);
- Similar correlations (i.e., positive) were found for VCAM-1 and the lymphocyte and monocyte ratio (although the latter relationship was no longer statistically significant—*p* = 0.1156).

## 4. Discussion

The aim of this study was to analyze the increase in VCAM-1 expression and the impact of exposure in a hyperbaric chamber on VCAM-1 in human blood serum. We believe that the results that have been obtained will allow for a better understanding of the biological changes that take place in our body during pressure changes at various depths, a deeper knowledge about the role of the VCAM-1 biomarker in our body and the possible impact of exposure in a hyperbaric chamber on human blood serum.

Madden and Laden [11], in their study, suggested that endothelial microparticles (MP) can be used as a decompression sickness (DCI) stress marker by assessing the antigenic markers of circulating MP that not only allow a specific origin, but also reflect endothelial integrity. According to these researchers, after endothelial disruption, the expression of adhesion molecules was expressed in accordance with the adopted configuration, and one such molecule, the VCAM-1 molecule, is an attractive marker due to the fact that it was only expressed on the activated endothelium, which was achieved after vascular trauma and is therefore a prognostic marker of a pro-inflammatory endothelium.

In this study, the statistical significance of differences in the distribution of VCAM-1 before and after exposure was analyzed. The difference between the VCAM-1 distribution before and after the exposure corresponding to a 30 m dive was on the limit of statistical significance (*p* = 0.0582). For most people, VCAM-1 was higher after exposure; on average, it was around 1.5 ng/mL, but there were also subjects who experienced a decrease in VCAM-1. The relative increase in VCAM-1 in some subjects was as high as 60% of the baseline value. Vince et al. [12] reported a significant increase in VCAM-1 positive microparticles (VCAM + MP), observed 1 h after diving using air compared to the non-divers group (*p* = 0.013), which was not observed after oxygen diving (*p* = 0.095).

The discussed study showed that diving at a greater depth had a much more pronounced effect on changes in VCAM-1 values. In all the subjects, VCAM-1 increased after exposure—at least by 0.8, and at most by 24.2 ng/mL. The average change was about 5.9 ng/mL. The changes in VCAM-1, as a result of diving to a depth of 60 m, were highly statistically significant. A study by Bao et al. [13] found that diving caused significantly reduced VCAM-1 levels.

Our research showed that the relative increase in VCAM-1 compared to the baseline value was as high as 80%, i.e., VCAM-1 after diving almost doubled in some subjects. A study performed by Zhang et al. [14] showed that VCAM-1 levels increased post-decompression in DCI rats.

In the presented study, a comparison of VCAM-1 distribution among divers before and after exposure to a 30 m and 60 m dive and the non-divers group was performed.

The distribution of the results obtained before and after exposure to the conditions corresponding to a 30 m dive did not statistically significantly differ from the distribution of results in the non-divers group. The research showed that there were statistically significant differences between the results obtained after exposure to conditions of a 60 m dive and the values measured for the non-divers group (*p* = 0.0494). VCAM-1 is expressed exclusively on the activated endothelium following vascular insult, and therefore, is a marker of a proinflammatory endothelium, as a study by Bao et al. [13] showed.

Research by Vince et al. [12] in which the VCAM + MP was quantified before diving (09:00 a.m. and 1:00 p/m.) and after diving (+1, +3 and +15 h), showed that both for diving with air and oxygen, and compared to control samples collected from the same subjects, VCAM + MP showed a similar trend in all the experiments. However, both dives resulted in a change in endothelial status as measured by VCAM + MP. A significant increase in VCAM + MP was observed 1 h after diving using air compared to the controls (*p* = 0.013), which was not observed after oxygen diving (*p* = 0.095). The researchers [12] observed an increase in the circulating VCAM + MP population after simulated diving with both compressed air and oxygen, compared to their non-dive controls taken at the same time of day. Due to its expression only on the activated endothelium, VCAM + MP can be used as a sensitive marker of endothelial function/dysfunction. Researchers hypothesized that the increase in circulating VCAM + MP could reflect changes in the state of the endothelium and could be potentially used as a biomarker of sensitivity to, for example, decompression sickness, when vascular mechanisms are involved [12].

This research showed that the results of BCC after the exposure of the subjects in the divers and non-divers group to conditions corresponding to a 30 m dive were not correlated with values of VCAM-1. However, the leukocyte count increased in a statistically significant manner after exposure to a 60 m dive, and exposure to conditions corresponding to a 30 m dive did not affect the unequivocally targeted change in the leukocyte count, which could be explained by an insufficient number of subjects in the divers group. A study performed by Glavas et al. [15] showed that the microbubbles produced during the decompression process induce endothelial damage and affect leukocyte mobilization.

In our study, the hemoglobin level decreased in most divers after exposure to diving conditions at a depth of 30 m and this effect should be considered as not accidental (*p* = 0.0098), and after a stronger exposure—to conditions corresponding to a depth of 60 m—such effects were not observed. The scale of the decrease in hemoglobin levels was small—only 0.2 g/dL on average; therefore, it was not a change affecting the health of the divers. Several studies have reported an altered hematological status and hemoglobin reduction after saturation diving [16,17,18,19].

A decrease in the number of neutrophils in the blood in our study is unlikely to indicate inflammation. Most likely, these results decrease from the fact that microbubbles are treated as hostile pathogens by neutrophils; as a result of the so-called oxygen burst (respiratory burst), microbubbles are eliminated, and thus the neutrophil cells are destroyed. This would confirm the increased generation of reactive oxygen species and the intensification of oxidative stress (we also observed an increase in oxidative stress in our volunteers).

More statistically significant relationships existed between the changes in the BCC parameters and the changes in VCAM-1 after exposure to conditions corresponding to a 60 m dive in the non-divers group. The relationships that occurred were as follows: higher increments of VCAM-1 were associated with a greater increase (in some cases a smaller decrease) in MCH and MCHC—these were relationships of average strength and statistically significant; quite a strong correlation was found between changes in neutrophils count and changes in VCAM-1—the greater the increase in the number of neutrophils is, the smaller the increase in VCAM-1 is. 

Our study showed that the number of blood platelets decreased in a statistically significant (*p* = 0.0382) manner after exposure to the conditions corresponding to a dive to a depth of 60 m, and the level of neutrophils increased in a statistically significant manner after exposure to conditions corresponding to a dive to a depth of 60 m. Olszański et al. [20] demonstrated in his study that the diving technology employed did not generate substantial changes in the examined parameters of blood in divers, and the increase in neutrophils, blood platelets and the fibrinogen concentration in the blood plasma immediately after diving is of a temporary character, being a typical reaction observed during diving.

In turn, the increase in the number of lymphocytes after exposure to conditions corresponding to a 60 m dive correlated with the increase in VCAM-1 (R = 0.54; *p* = 0.0304); similar (i.e., positive) correlations apply to VCAM-1 and the lymphocyte ratio. This research also showed that those few correlations that were close to the level of statistical significance, and in one case statistically significant, occurred between BCC and VCAM-1 after exposure to conditions corresponding to a 60 m dive in the divers group. The relationships were as follows: there was a relationship between the neutrophils count and VCAM-1—the higher the number of neutrophils was, the lower the value of VCAM-1 was (this correlation was statistically significant—*p* = 0.0456; its strength was average—R = −0.51); a similar relationship was found between the number of leukocytes and VCAM-1 (it was slightly weaker and only close to the level of statistical significance); VCAM-1 was higher in the subjects with a higher lymphocyte ratio (R = 0.44)—this correlation was close to the level of statistical significance (*p* = 0.0848). In a study performed by Bao et al. [13], deep heliox diving caused a significant decrease in red blood cells (RBC) but had no significant effect on hemoglobin (HGB) levels. These changes can be explained by the oxidative damage-induced fragility of the RBC membrane. A study by Perovic et al. [21] showed that neutrophils increased, and monocytes decreased immediately after 30 m-depth compressed-diving using air. Since the number of intermediate cells in humans is small, the reduction in the percentage of intermediate cells may be a transient response to external stimuli. Obad at al. [22] have shown in their study that this may be caused by trans-endothelial migration due to altered vascular/endothelial function after diving. Sureda et al. [23] has shown in his study that scuba diving at 50 m deep for a total time of 35 min was enough to induce a post-diving neutrophil mobilization in normobaria, suggesting the initiation of an immune-like response, similar to that which occurs after an infection or an acute bout of exercise. Exactly these results could be expected, because VCAM-1 is a key cell adhesion molecule involved in inflammation that is closely implicated in various immunological disorders [24,25]. The VCAM-1 protein mediates the adhesion of neutrophils, monocytes, eosinophils and basophils to the vascular endothelium [14,26]. It is also active in the signal transduction of leukocytes and endothelial cells [24,27,28,29]. A study by Glavas et al. [15] showed that the absolute number of monocytes was slightly, but not significantly, increased after a dive, and this study suggests that biochemical changes induced by scuba diving primarily activate existing monocytes rather than increase the number of monocytes at a time of acute arterial endothelial dysfunction.

### Strengths and Limitations of the Study

A strength of this study was the well-matched divers and non-divers groups.

The limitations of the present study should be noted. Due to the small sample size, the present study has limited power. We believe that further research with a larger population is required and warranted. In searching for the relationship between the BCC results of blood counts and the values of VCAM-1 in the test group (conditions corresponding to a 30 m dive), the lack of a relationship with white blood cells can also be explained by the group of respondents being too small.

## 5. Conclusions

1. The results confirm our hypothesis that exposure to a hyperbaric chamber has an effect on VCAM-1 in blood serum in the divers group.
2. There is a correlation between the tested BCC and VCAM-1 before and after exposure in the chamber in the divers group.
3. We believe that exposure in a hyperbaric chamber may result in the activation of the endothelium.

## Figures and Tables

**Table 1:** 3 MW decompression tables.

Exposition	Depth	Bottom Time	Time to First Stop	Decompression Stops (mH_2_O)														Total Ascent Time			
				42	39	36	33	30	27	24	21	18	15	12	9	6	3				
				Time on the Stops (min)														Air		Oxygen	
	(mH_2_O)	(min)	(min)	Air									Air or (Oxygen)					(h)	(min)	(h)	(min)
30 m	33	35	3												6(3)	10(5)	16(8)		35		19
60 m	63	35	6							9	13	16	18(9)	22(11)	32(16)	47(24)	58(29)	3	41	*2*	13

**Table 2:** Demographic and occupational status of subjects.

Demographic and Occupational Status	Group				*p*
	Divers (N = 18)		Non-Divers (N = 14)		
	N	%	N	%	
**Sex**					0.0391 *
female	0	0.0%	3	21.4%	
male	18	100.0%	11	78.6%	
**Age (years)**	33.9 ± 6.6		33.0 ± 8.4		0.5521
**Place of residence**					0.0515
Rural	5	29.4%	0	0.0%	
municipality up to 50,000 inhabitants	8	47.1%	5	35.7%	
municipality 50,000–100,000 inhabitants	1	5.9%	1	7.1%	
municipality over 100,000 inhabitants	3	17.6%	8	57.1%	
**Education**					0.1739
Secondary	8	44.4%	3	21.4%	
Higher	10	55.6%	11	78.6%	
**Occupational seniority (years)**	10.4 ± 7.3		10.6 ± 7.8		0.9254
**Type of work**					0.0436 *
physical	15	83.3%	7	50.0%	
mental	3	16.7%	7	50.0%	
**Type of physical effort**					0.0043 **
Intensive	3	17.0%	2	14.3%	
Moderate	4	22.0%	2	14.3%	
Variable	3	17.0%	0	0.0%	
Aerobic	0	0.0%	8	57.1%	
None	8	44.0%	2	14.3%	

Abbreviations: *—*p* < 0.05; **—*p* < 0.01.

**Table 3:** Subject lifestyle.

Lifestyle	Group				*p*
	Divers (N = 18)		Non-Divers (N = 14)		
	N	%	N	%	
**Drinking coffee**					0.3365
None	6	33.3%	4	28.6%	
Once a day	4	22.2%	3	21.4%	
Twice a day	6	33.3%	2	14.3%	
More	2	11.1%	5	35.7%	
**Tobacco smoking**					0.7876
Yes	2	11.1%	2	14.3%	
No	16	88.9%	12	85.7%	
**Self-assessment of physical condition**					0.0842
Ideal	1	5.6%	4	28.6%	
Good	17	94.4%	8	57.1%	
Intermediate	0	0.0%	1	7.1%	
Hard to tell	0	0.0%	1	7.1%	

**Table 4:** 95% confidence intervals for the average level of VCAM-1 parameter measured in four tested situations in the divers group.

VCAM-1 (ng/mL)	95% CI
Before exposure—30 m	(10.7; 20.5)
After exposure—30 m	(12.3; 21.9)
Before exposure—60 m	(9.2; 16.4)
After exposure—60 m	(12.8; 24.6)

Abbreviations: CI—confidence interval; VCAM-1—vascular cell adhesion molecule-1.

**Table 5:** Distribution of VCAM-1 before and after the exposure corresponding to a 30 m and 60 m dive in the divers group.

VCAM-1 [ng/mL] 30		**M**	**Me**	**SD**	**Min.**	**Max.**
	Before dive	15.6	14.6	9.9	5.6	46.1
	After dive	17.1	16.6	9.6	6.7	41.5
	Change (*p* = 0.0582)	1.5	1.1	2.8	−4.6	6.6
VCAM-1 [ng/mL] 60	Before dive	12.8	12.4	7.2	5.0	31.1
	After dive	18.7	17.5	11.8	6.7	55.3
	Change (*p* = 0.0002 ***)	5.9	4.2	5.3	0.8	24.2

Abbreviations: M—mean; Me—median; SD—standard deviation; Min.—minimum; Max.—maximum; ***—*p* < 0.001.

**Table 6:** Values of descriptive statistics characterizing the distribution of VCAM-1 in the compared groups.

VCAM-1 (ng/mL)	Group										*p*
	Divers (N = 18)					Non-Divers (N = 14)					
	$\bar{x}$	Me	*s*	Min	Max	$\bar{x}$	Me	*s*	Min	Max	
before exposure—30 m	15.6	14.6	9.9	5.6	46.1	11.1	9.9	4.5	7.2	25.0	0.2666
after exposure—30 m	17.1	16.6	9.6	6.7	41.5						0.1251
before exposure—60 m	12.8	12.4	7.2	5.0	31.1						0.8662
after exposure—60 m	18.7	17.5	11.8	6.7	55.3						0.0494 *

Abbreviations: *—*p* < 0.05.

**Table 7:** Descriptive statistics characterizing the distribution of leukocytes and hemoglobin counts in individual tests, showing the significance of changes between tests completed before and after exposure.

		M	Me	SD	Min.	Max.
Leukocytes [G/L]	Before dive 30 m	6.20	6.19	1.05	4.23	9.03
	After dive 30 m	6.43	6.22	1.23	4.42	8.87
	Change (*p* = 0.2485)	0.23	0.18	0.74	−0.76	1.56
	Before dive 60 m	5.95	6.01	0.96	3.70	7.56
	After dive 60 m	7.10	7.26	1.28	4.55	9.33
	Change (*p* = 0.0004 ***)	1.17	0.94	0.85	0.12	3.20
Hemoglobin [g/dL]	Before dive 30 m	15.2	15.3	0.8	14.0	16.8
	After dive 30 m	15.0	15.0	0.9	13.4	16.8
	Change (*p* = 0.0098 **)	−0.2	−0.2	0.3	−1.0	0.4
	Before dive 60 m	15.2	15.2	0.8	13.8	16.4
	After dive 60 m	15.0	15.1	0.8	13.8	16.3
	Change (*p* = 0.1673)	−0.1	0.0	0.3	−0.9	0.3

Abbreviations: G/L—billion per liter; M—mean; Me—median; SD—standard deviation; Min.—minimum; Max.—maximum; **—*p* < 0.01; ***—*p* < 0.001.

## Data Availability

Data are available upon reasonable request.
